# A case report of renal papillary necrosis due to tuberculosis—CT urogram and static MR urogram findings

**DOI:** 10.1259/bjrcr.20150438

**Published:** 2017-01-07

**Authors:** Denver Steven Pinto, Arun George, Nidhi Kumar, V Ravi Hoisala

**Affiliations:** Department of Radiodiagnosis, St Johns Medical College, Koramangala, Bangalore, India

## Abstract

The urinary tract is a common site of tuberculosis, which causes significant morbidity in the form of chronic renal disease. T uberculosis is not only common in developing countries but with the spurt in the number of immune-suppressed patients and the increasing incidence of drug -resistant strains, an increase in the number of patients suffering from genitourinary tuberculosis is expected even in developed countries. Genitourinary tuberculosis occurs owing to haematogenous dissemination of tubercular bacilli. Urinary tract tuberculosis can result in complications such as ureteric stricture, chronic pyelonephritis and papillary necrosis, resulting in compromised renal function. This renal compromise makes it prudent to avoid contrast- enhanced studies if other alternatives are available. There is a dearth of-cases of papillary necrosis reported on static MR urogram. The authors report a case of tuberculosis complicated by papillary necrosis on both CT urogram and static MR urogram.

## Background

Urogenital tuberculosis is the most common cause of extrapulmonary tuberculosis, affecting about 27% of patients suffering from it.^1^ Tuberculosis is not only common in developing countries; but with the spurt in the number of immune-suppressed patients and the increasing incidence of drug-resistant strains, an increase in the number of patients suffering from genitourinary tuberculosis is expected even in developed countries.^1^ Genitourinary tuberculosis occurs owing to haematogenous dissemination of tubercular bacilli.^1,2^ Discussed below is a patient from a low socioeconomic background who presented for evaluation of flank pain and weight loss. It is important to suspect and diagnose tuberculosis on imaging to direct the clinician to perform appropriate investigations such as culture for Mycobacteria. Appropriate treatment is necessary to prevent complications such as ureteric strictures, putty kidney, thimble bladder and tubercular renal abscesses.

Many patients with renal tuberculosis have compromised renal function. This makes it prudent to avoid the use of contrast in such patients. This case report aims to show that static MR urogram can be used to make the diagnosis of renal tuberculosis without the risks of contrast or radiation dose administration. This case report describes the findings of renal tuberculosis with papillary necrosis on static MR urogram.

## Clinical presentation

A 60-year-old male presented with left flank pain and weight loss. Because of clinical suspicion of urolithiasis the patient was referred for imaging. On clinical examination there was no flank tenderness. A few enlarged cervical and inguinal lymph nodes were found.

Clinical and Laboratory Evaluation: Routine urine analysis showed albuminuria 2+, numerous leucocytes in urine and negative nitrite test. On urine microscopy 227 RBCs/hpf, 314 WBCs/hpf and 239 bacteria/hpf were found (hpf = high power field). However, there was no growth on bacterial culture. This was indicative of sterile pyuria. A diagnosis of renal tuberculosis was considered with a differential diagnosis of chronic pyelonephritis.

## Imaging

**Figure 1.**
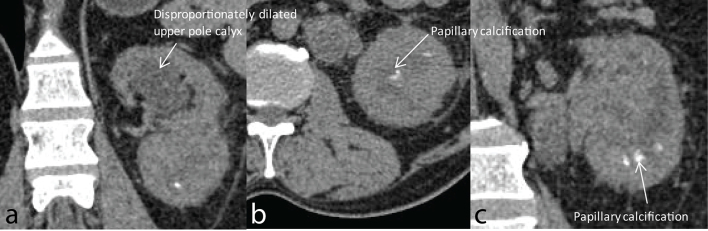
(a–c) Plain CT images axial and coronal reformats (a) showing disproportionately dilated upper pole calyces with perinephric fat stranding; (b,c) showing papillary calcifications in the lower pole.

**Figure 2.**
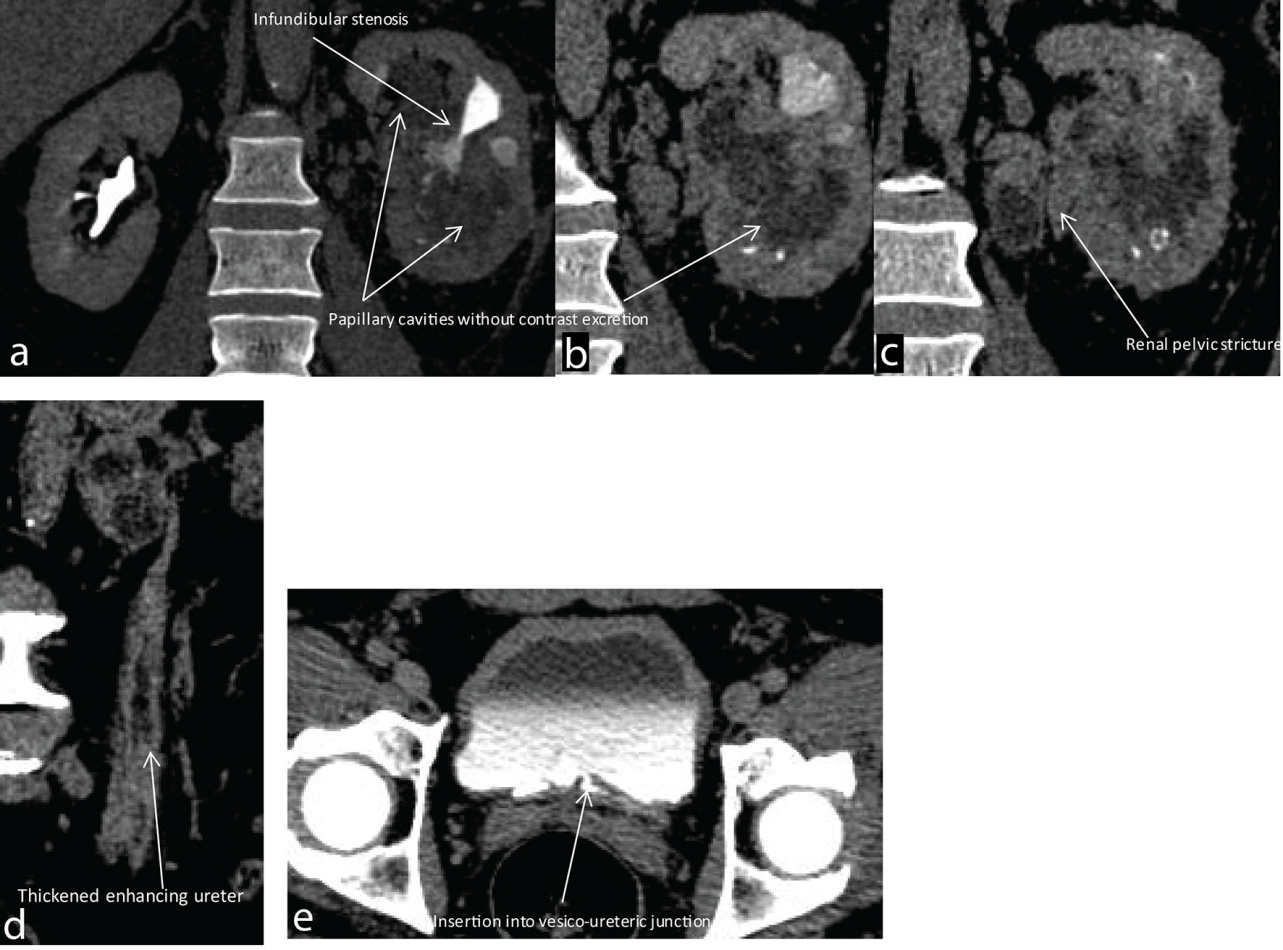
(a–d) Coronal reformats of CT urogram images showing (a,b) papillary cavities with distorted calyces in the upper and lower poles without opacification by contrast. Infundibular stenosis with a dilated triangular-shaped calyx is seen suggesting a papillary cavity with excreting calyces or a papillary cavity showing communication with the calyceal system. (c) Also seen is the narrowed lumen of the renal pelvis with wall thickening suggesting a renal pelvic stricture. A necrotic hilar lymph node is also seen in these images. (d) Coronal reformat showing irregularly thickened enhancing walls of the ureter. (e) - -, The terminal ureter and the vesicoureteral junction shows wall thickening.

**Figure 3.**
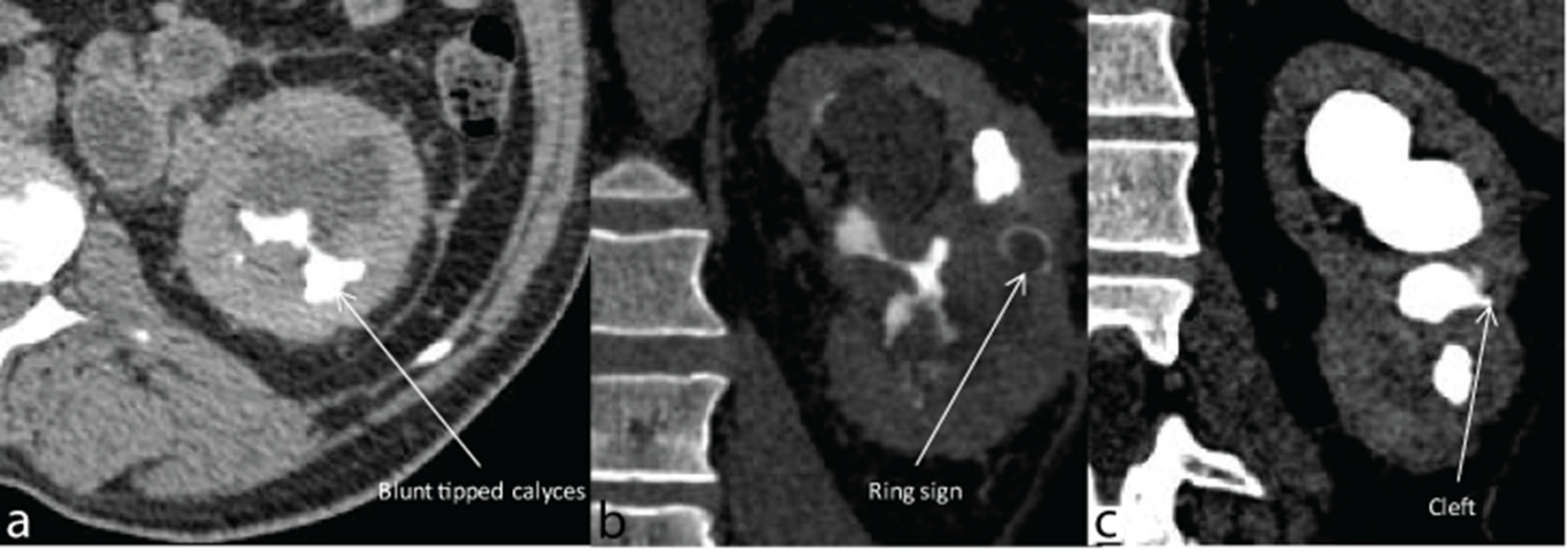
(a–c) Axial and coronal CT sections showing different signs of papillary necrosis: (a) blunt-tipped calyces in the right lower pole; (b) a ring sign, where a ring-like contrast opacification is noted around centrally non-opacified sloughed off papilla within the interpolar calyx. (c) Clefting in the middle interpolar calyx. In this coronal section pooling of contrast in dilated upper pole calyces is seen.

**Figure 4. f4:**
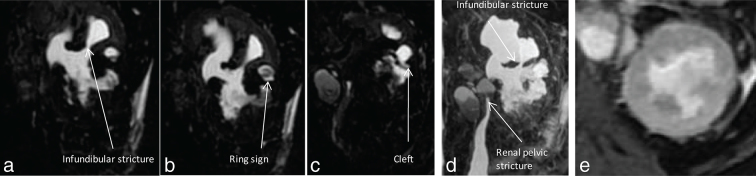
(a–c) showing coronal slices of a 3D magnetic resonance cholangiopancreatography sequence and (d) showing the maximum intensity projection of the 3D magnetic resonance cholangiopancreatography sequence of the left kidney. Seen in (a) is the infundibular stenosis with the triangular papillary cavity corresponding to the CT urogram in Figure 2a. (b) shows a ring sign corresponding to the CT urogram image in Figure 3b with distorted and irregular lower pole calyces. (c) shows a cleft in the interpolar calyx. Also seen in this image is the renal hilar lymph node. (d) shows the renal pelvic and interpolar infundibular stricture with disproportionately dilated upper and lower pole calyces, with irregular contour of the lower pole calyces. [Fig f4] (e) shows *T*_2_ hyperintensity of the renal parenchyma.

**Figure 5.**
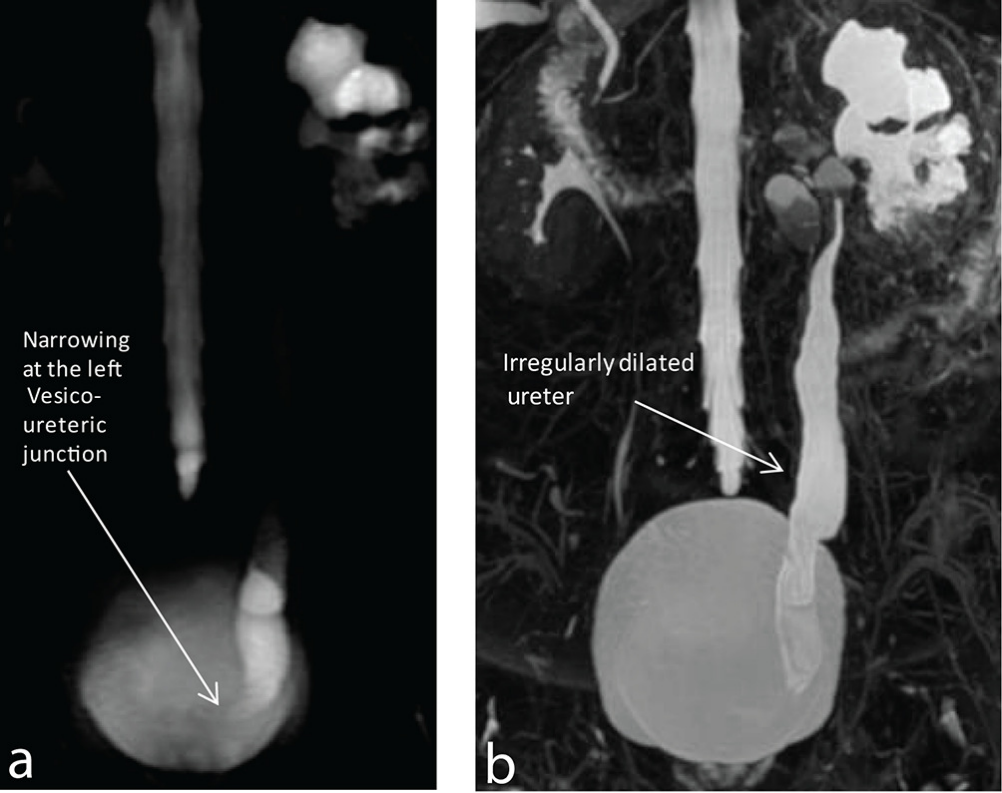
(a,b) Thick slab magnetic resonance cholangiopancreatography and maximum intensity projection of thin slab (3 mm) 3D magnetic resonance cholangiopancreatography sequence show irregular dilation of the- - lower ureter, with abrupt narrowing at its insertion into the vesicoureteral junction.

Initial evaluation on plain CT showed disproportionately dilated upper pole calyces and urothelial thickening with narrowing of the pelvis with papillary calcifications in the lower pole. Significant perinephric and periureteric fat stranding with locoregional lymphadenopathy was noted (Figure 1). This was followed up by a CT urogram. CT urogram showed multiple filling defects within the calyceal system with a papillary cavity in the upper and lower poles with calyceal distortion and infundibular-pelvic stenosis. Also, the ureter showed thickened and enhancing walls (Figure 2). The features of disproportionate calyceal dilation with papillary cavities, infundibular-pelvic stenosis and calcifications with necrotic locoregional adenopathy are suggestive of an infective aetiology such as tuberculosis. Other signs of papillary necrosis such as blunt-tipped calyces and a ring sign—where contrast is seen surrounding a centrally non-opacified calyx—are seen (Figure 3). This was followed by a static MR urogram to show that the findings demonstrated on a CT urogram with contrast administration could also be demonstrated without contrast administration on an MR urogram. The following MR sequences were used—axial 2D FIESTA FATSAT (TR 3.60 ms, TE 1.55 ms. Slice thickness 8 mm, ET 1, matrix size 320 × 224), Coronal 2D FIESTA FATSAT (TR 3.60 ms, TE 1.56 ms. Slice thickness 5 mm, ET 1, matrix size 192 × 288), 3D magnetic resonance cholangiopancreatography (MRCP) RTr ASSET (TR 3750 ms, TE 383 ms. Slice thickness 1.6 mm, ET 64, matrix size 256  × 256), diffusion-weighted imaging axial (TR 6250 ms, TE 93.50 ms. Slice thickness 8 mm, ET 1, matrix size 128  × 128), diffusion-weighted imaging coronal (TR 5825 ms, TE 92.20 ms. Slice thickness 5 mm, ET 1, 128 × 128) and thick slab MRCP ASSET coronal sequence(TR 2566 ms, TE 1202 ms. Slice thickness 60 mm, ET 1, 384 × 256). MR urogram showed the filling defects within the calyces including the signs of papillary necrosis found on the CT urogram such as calyceal filling defects, blunt-tipped calyces, the ring sign and clefts (Figure 4). Thick and thin slab 3D MRCP sequences showed the calyceal findings of papillary necrosis with a dilated ureter with narrowing at its insertion suggestive of a stricture. The distal ureteric stricture was seen better on MRI (Figure 5).

## Discussion

The most common extrapulmonary site of tuberculosis is the urinary tract, with almost all cases resulting from haematogenous seeding. Despite the presumed route of spread from the lungs to the kidney, less than half the patients who have genitourinary tuberculosis have abnormal chest radiographs.^2^

The tubercular bacilli form granulomas within the kidney and remain indolent for many years. Tuberculosis has a predilection for the upper and lower poles of the kidney. Imaging findings of renal tuberculosis result from the combination of papillary necrosis and parenchymal destruction. These papillary lesions caseate and cavitate, forming ulcerocavernous lesions as they erode into the pelvicalyceal system. Extensive papillary necrosis may develop with the formation of cavities and destruction of the renal parenchyma. They may also rupture into the collecting system, or cause parts of the papillae to become necrotic and slough. The collecting system shows thickening, ulceration and fibrosis, often with stricture formation. Along with papillary necrosis, the above spectrum is highly suggestive of tuberculosis.^3–5^

In a comprehensive review of the pathophysiology and imaging of renal tuberculosis, Merchant et al^3,5^ stated that pelvic- infundibular strictures, papillary necrosis, cortical low-attenuation masses, scarring and calcification may be seen in other conditions, but the combination of three or more of these findings is highly suggestive of tuberculosis, even in the absence of documented pulmonary disease.

However, CT is still limited in the identification of granulomas of about 3 mm size, minimal urothelial thickening and subtle papillary necrosis.^6^ Few studies have reported the features of genitourinary tuberculosis on MRI. Loss of interface between the infection and the adjacent renal parenchyma, the surrounding tissue oedema, the asymmetric perinephric fat stranding and the thickening of Gerota’s fascia may be clues indicating that the focal pyelonephritis has a tuberculous origin.^5^

Urine culture was done for the patient, which showed growth of *Mycobacterium tuberculosis*. The patient was started on antitubercular chemotherapy and put on follow-up with a plan to perform ureteric stenting. Cystoscopy confirmed the finding of a thickened and narrowed left ureteric opening, which was characteristic of tuberculosis.

In this case report, the authors describe the calyceal findings of urinary tract tuberculosis causing papillary necrosis on both CT and static MR urogram with greater emphasis on the calyceal and ureteral findings of the disease. The finding of terminal ureteric narrowing was more easily seen on MR urogram. The findings seen on contrast-enhanced CT urogram could also be seen on static MR urogram without administration of contrast. Thus, in patients with renal dysfunction static MR urogram can be used instead of contrast-enhanced CT urogram.

## Learning points

1. MR urogram can detect the calyceal and ureteric findings of tuberculosis with papillary necrosis without contrast administration.
2. The signs of papillary necrosis are blunting of calyces, filling defects in the calyces, ring sign, presence of clefts and infundibular pelvic stenosis, which can be seen both on CT and MR urogram.
3. MRI may be better than CT for detecting terminal ureteric strictures.

## Consent

Informed consent to publish this case (including images and data) was obtained and is held on record.
